# Cusp Density and Commensurability of Non-arithmetic Hyperbolic Coxeter Orbifolds

**DOI:** 10.1007/s00454-022-00455-z

**Published:** 2022-11-25

**Authors:** Edoardo Dotti, Simon T. Drewitz, Ruth Kellerhals

**Affiliations:** Department of Mathematics, University of Fribourg, 1700 Fribourg, Switzerland

**Keywords:** Hyperbolic orbifold, Coxeter group, Commensurability, Vinberg space, Non-arithmeticity, Cusp density, 22E40, 57M50, 11E88, 20F55

## Abstract

For three distinct infinite families $(R_m)$, $(S_m)$, and $(T_m)$ of non-arithmetic 1-cusped hyperbolic Coxeter 3-orbifolds, we prove incommensurability for a pair of elements $X_k$ and $Y_l$ belonging to the same sequence and for most pairs belonging two different ones. We investigate this problem first by means of the Vinberg space and the Vinberg form, a quadratic space associated to each of the corresponding fundamental Coxeter prism groups, which allows us to deduce some partial results. The complete proof is based on the analytic behavior of another commensurability invariant. It is given by the cusp density, and we prove and exploit its strict monotonicity.

## Introduction

Let ${\mathbb {H}}^n$ be the real hyperbolic space of dimension $n\ge 2$ with its isometry group ${{\,\textrm{Isom}\,}}{\mathbb {H}}^n$. The quotient space $O^n={\mathbb {H}}^n/\Gamma$ of ${\mathbb {H}}^n$ by a discrete subgroup $\Gamma \subset {{\,\textrm{Isom}\,}}{\mathbb {H}}^n$ of finite covolume is a hyperbolic *n*-orbifold. By Selberg’s lemma, each orbifold is finitely covered by a manifold.

In low dimensions, there are different ways to construct hyperbolic orbifolds and manifolds. In this work, we consider only non-compact space forms of dimension three. The arithmetic constructions in the orientable context are related to Bianchi groups, that is, to Kleinian groups of the form ${{\,\textrm{PSL}\,}}(2,\mathcal O_d)\subset {{\,\textrm{PSL}\,}}(2,{\mathbb {C}})$ where ${{\mathcal {O}}}_d$ is the ring of integers in the field ${\mathbb {Q}}(\sqrt{-d})$. A topological way is to look at knot and link complements in ${\mathbb {S}}^3$ that carry a hyperbolic structure. For $n=3$, we are interested in cusped hyperbolic Coxeter *n*-orbifolds arising as quotients by hyperbolic Coxeter groups, that is, by discrete groups generated by finitely many reflections in hyperplanes of ${\mathbb {H}}^n$. A fundamental polyhedron for a hyperbolic Coxeter group is a so-called Coxeter polyhedron *P* given by a convex polyhedron all of whose dihedral angles are integral submultiples of $\pi$. We assume that *P* as convex hull of finitely many ordinary or ideal points has at least one vertex on the ideal boundary $\partial {\mathbb {H}}^n$. These orbifolds form a very natural and important family of cusped hyperbolic space forms that include orbifolds of small volume in various dimensions up to $n=18$ (see [12, 13]).

In contrast to higher dimensions, there are infinitely many distinct Coxeter 3-orbifolds, and some of them are intimately related to Bianchi orbifolds or knot and link complements as described above (see [1, Sect. 7], [16, Sect. 3], and Remark 4.3, for example). In order to obtain a survey about the variety of cusped hyperbolic orbifolds, we study them up to commensurability. Two hyperbolic *n*-orbifolds are commensurable if they have a common finite-sheeted cover, which means that their fundamental groups are commensurable (in the wide sense). Notice that properties such as arithmeticity and cocompactness are stable with respect to commensurability. As an example, the arithmetic 1-cusped Gieseking manifold $M_G$, arising by side identifications of an ideal regular tetrahedron $S^{\infty }_{reg }$, has a double cover homeomeorphic to the Figure Eight knot complement, and the fundamental group of $M_G$ is commensurable to the Coxeter group associated to $S^{\infty }_{reg }$ as well as to the Bianchi group ${{\,\textrm{PSL}\,}}(2,{{\mathcal {O}}}_3)$. In the case of arithmetic hyperbolic 3-orbifolds, there is a well developed and very satisfactory theory about the commensurability of Kleinian groups (see [15, 18]). In the case of non-arithmetic hyperbolic 3-manifolds, there is a general algorithm for deciding about their commensurability in terms of horosphere packings and canonical cell decompositions (see [6]).

In this work, we study commensurability of infinitely many distinct non-arithmetic 1-cusped hyperbolic Coxeter 3-orbifolds. More precisely, we consider three infinite sequences $(R_m)$, $(S_m)$, and $(T_m)$ describing simultaneously certain Coxeter prisms (see Fig. 3), their reflection groups and the related Coxeter orbifolds, and defined via their Coxeter graphs as below (for details, see Sect. 2.2). These Coxeter orbifolds are 1-cusped and, for $m\ge 7$, non-arithmetic.

**Fig. 1:**
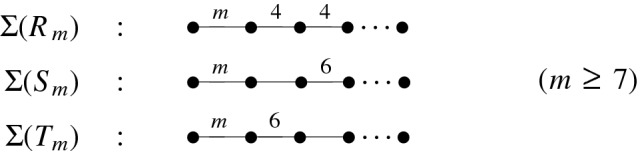
The three sequences $(R_m)$, $(S_m)$, and $(T_m)$ of Coxeter prism groups

The aim of this work is to prove the following result.

### Theorem

For an integer $m\ge 7$, consider the three sequences of non-arithmetic 1-cusped hyperbolic Coxeter 3-orbifolds induced by $(R_m)$, $(S_m)$, and $(T_m)$ according to Fig. 1. Then

- two distinct elements $X_k$ and $X_l$ belonging to the same sequence are incommensurable;
- each element $R_k$ is incommensurable with any element $X_l$ not belonging to the sequence $(R_m)$;
- the elements $S_k$ and $T_l$ are incommensurable for $k\ge l$.

For the proof of our Theorem, we first exploit some new commensurability conditions for pairs of hyperbolic Coxeter groups such as those given by Fig. 1. These necessary conditions rely upon the Vinberg space and the Vinberg form related to an arbitrary hyperbolic Coxeter group, and they were recently established by the first author [3, 4]. This investigation leads to first yet incomplete conclusions. A complete proof of our Theorem is based on the study of the cusp density $\delta (X_m)$ of the orbifold $X_m$. The quantity $\delta (X_m)$ is given by the ratio of the volume of the maximal (embedded) cusp in $X_m$ to the total volume of $X_m$ and forms a commensurability invariant in the context of non-arithmetic 1-cusped hyperbolic orbifolds (it is, however, not a complete invariant; see [6, Sect. 1]). For each of the sequences $(R_m)$, $(S_m)$, and $(T_m)$, we derive explicit formulas for $\delta (X_m)$ and prove and exploit the strict monotonicity of their cusp density as a function of *m*. These monotonicity properties are not of uniform nature but help us in a crucial way to provide a coherent and complete proof of the above theorem.

This work is structured as follows. In Sect. 2, we review the basic concepts of hyperbolic Coxeter groups, Coxeter polyhedra and their graphs, and present Vinberg’s arithmeticity criterion (see Sect. 2.2). For prisms in ${\mathbb {H}}^3$ giving rise to the Coxeter realisations $(R_m)$, $(S_m)$, and $(T_m)$ and the related cusped orbifolds, we recapitulate a volume formula in terms of the Lobachevsky function. In this way, the cusp density as presented in Sect. 2.1 takes a more concrete analytic form. In Sect. 3, we introduce the notion of Vinberg’s quadratic space and use it to formulate the commensurability conditions for a pair of hyperbolic Coxeter groups in Theorem 3.1. Its impact for subfamilies of groups belonging to the sequences $(R_m)$, $(S_m)$, and $(T_m)$ form our first conclusions presented at the end of the section. In Sect. 4, we treat the cusp density $\delta (X_m)$ from a polyhedral point of view and look at the cusp density function for the maximal cusp in a corresponding prism $R'(\alpha ,\beta )\subset {\mathbb {H}}^3$ defined by two angular parameters $\alpha ,\beta$ with $0<\alpha +\beta <{\pi }/{2}$ (see Fig. 3). A key technical result to prove strict monotonicity of $\delta (X_m)$ is Proposition 4.2 that describes the cusp volume in $R'(\alpha ,\beta )$ in terms of the sign of $\cos \alpha -\sqrt{2}\sin \beta$. In Remark 4.3, we consider the case $\cos \alpha =\sqrt{2}\sin \beta$ for $\alpha ={\pi }/{m}$, $m\in {\mathbb {N}}_{\ge 3}$, and discuss briefly the close connection of the prism $R'({\pi }/{m},\beta )$ with Thurston’s polyhedral model for the *m*-chain link complement ${\mathbb {S}}^3\setminus C_m$. Finally, and based on Schläfli’s differential expression for hyperbolic volume, we are able to provide a complete and self-contained proof of our theorem.

## Commensurability of Hyperbolic Orbifolds

Let $\Gamma <{{\,\textrm{Isom}\,}}{\mathbb {H}}^n$ be a hyperbolic lattice, that is, $\Gamma$ is a discrete group of isometries acting on ${\mathbb {H}}^n$ with a fundamental polyhedron $P\subset {\mathbb {H}}^n$ of finite volume. The latter property describes $\Gamma$ as being cofinite. The quotient $O^n={\mathbb {H}}^n/\Gamma$ is a hyperbolic *n*-orbifold whose volume is given by the volume of *P*, also denoted by $covol _n(\Gamma )$. Two such orbifolds $O^n_1$ and $O^n_2$ are *commensurable* if they have a common finite sheeted cover. Equivalently, their fundamental groups $\Gamma _1,\Gamma _2\subset {{\,\textrm{Isom}\,}}{\mathbb {H}}^n$ are commensurable in the sense that there is an element $\gamma \in {{\,\textrm{Isom}\,}}{\mathbb {H}}^n$ such that $\Gamma _1\cap \gamma \Gamma _2\gamma ^{-1}$ has finite index in both $\Gamma _1$ and $\gamma \Gamma _2\gamma ^{-1}$. The commensurability property for groups in ${{\,\textrm{Isom}\,}}{{\mathbb {H}}}^n$ yields an equivalence relation preserving characteristics such as discreteness, cofiniteness and arithmeticity. In this context, a fundamental result of Margulis (see [22, Chap. 6], for example) states that a hyperbolic lattice $\Gamma \subset {{\,\textrm{Isom}\,}}{{\mathbb {H}}}^n$, $n\ge 3$, is non-arithmetic if and only if its commensurator

2.1 $$\begin{aligned} {{\,\textrm{Comm}\,}}(\Gamma )=\{\gamma \in {{\,\textrm{Isom}\,}}{{\mathbb {H}}}^n\mid \Gamma \text { and } \gamma \Gamma \gamma ^{-1} \text { are commensurable}\} \end{aligned}$$

is a hyperbolic lattice, and containing $\Gamma$ as a subgroup of finite index. In particular, ${{\,\textrm{Comm}\,}}(\Gamma )$ is the (unique) maximal group commensurable with a non-arithmetic hyperbolic lattice $\Gamma$.

### Cusp Density of a Non-Compact Hyperbolic Orbifold

In the sequel, we study commensurability of different infinite families of *cusped* non-arithmetic hyperbolic 3-orbifolds. A *cusp* *C* of an orbifold $O^n={{\mathbb {H}}}^n/\Gamma$ is a connected subset of $O^n$ that lifts to a set of horoballs with disjoint interiors in ${{\mathbb {H}}}^n$. The set *C* gives rise to an ideal vertex $q\in \partial {{\mathbb {H}}}^n$ of a fundamental polyhedron for $\Gamma$, and *C* is of the form $B_q/\Gamma _q$ where $B_q\subset {{\mathbb {H}}}^n$ is a horoball internally tangent to *q* and where $\Gamma _q<\Gamma$ is the stabiliser of *q*. By Bieberbach’s theory, $\Gamma _q$ is a crystallographic group acting discretely and cocompactly by Euclidean isometries on the horosphere $\partial B_q$ containing a translation lattice of rank 2.

Suppose that a hyperbolic orbifold $O^n$ has precisely one cusp *C*, and that *C* is *maximal*, that is, there is no cusp of $O^n$ containing *C*. This means that *C* is tangent to itself at one or more points. The ratio

2.2 $$\begin{aligned} \delta (O^n)=\delta (C)=\frac{{{\,\textrm{vol}\,}}_n(C)}{{{\,\textrm{vol}\,}}_n(O^n)} \end{aligned}$$

is called the *cusp density* of $O^n$ (and given by *C*). The numerator ${{\,\textrm{vol}\,}}_n(C)$ of $\delta (C)$ can be computed in terms of the volume of a Euclidean fundamental polyhedron $P_q$ for the group $\Gamma _q$ as follows. Pass to the upper half space model for ${{\mathbb {H}}}^n$ in ${{\mathbb {R}}}^n_+$ where infinitesimal arc length is given by $ds={dx}/{x_n}$. Suppose without loss of generality that $q=\infty$ and that the bounding horosphere $\partial B_{\infty }$ is the hyperplane $\{x_n=1\}$ at distance 1 from the ground space ${{\mathbb {R}}}^{n-1}$. Then, the volume of the cusp *C* is given by (see also [11, Sect. 3])

2.3 $$\begin{aligned} {{\,\textrm{vol}\,}}_n(C)={{\,\textrm{vol}\,}}_{n-1}(P_{\infty })\int \limits _1^{\infty }\frac{dx_n}{x_n^n}=\frac{{{\,\textrm{vol}\,}}_{n-1}(P_{\infty })}{n-1}. \end{aligned}$$

The following result is an easy consequence of the above concepts and facts and will play a crucial role (see [18, Prop. 1], [6, Sect. 2]).

#### Proposition 2.1

The cusp density is a commensurability invariant for non-arithmetic 1-cusped hyperbolic orbifolds.

### Hyperbolic Coxeter Groups and Coxeter Orbifolds

Interpret hyperbolic space in the hyperboloid model ${{\mathcal {H}}}^n$ as a subset of ${{\mathbb {R}}}^{n+1}$ equipped with the Lorentzian form $q(x)=-x_0^2+x_1^2+\ldots +x_n^2$ as usual. The group of isometries ${{\,\textrm{Isom}\,}}{{\mathbb {H}}}^n$ is given by the group $\hbox {O}^+(n,1)$ of positive Lorentzian matrices.

For $N\ge n+1$, let $\Gamma \subset {{\,\textrm{Isom}\,}}{{\mathbb {H}}}^n$ be a hyperbolic lattice generated by finitely many reflections $s_i$ in hyperplanes $H_i=e_i^{\perp }$, $1\le i\le N$, of ${{\mathcal {H}}}^n$. As a consequence, the vectors $e_1,\ldots ,e_N$ contain a Lorentzian basis of ${{\mathbb {R}}}^{n+1}$ which we suppose to be of Lorentzian norm 1. Consider the convex polyhedron

2.4 $$\begin{aligned} P\,=\!\bigcap _{1\le i\le N}\!H_i^{-} \end{aligned}$$

of closed half-spaces $H_i^-\subset {{\mathcal {H}}}^n$ with outer normal vectors $e_i$. The polyhedron *P* is a *Coxeter polyhedron*, that is, all the dihedral angles of *P* are of the form $\pi /m$ for an integer $m\ge 2$. In this way, the group $\Gamma$ is a *hyperbolic Coxeter group* and a geometric representation of an abstract Coxeter group in the group $\hbox {O}^+(n,1)$. The associated orbit space of $\Gamma$ is called a *hyperbolic Coxeter n-orbifold*. The theory of hyperbolic Coxeter groups and orbifolds has been developed essentially by Vinberg (see [5, 20, 21] for classification results and further references).

Associated to *P* and $\Gamma$ is the Gram matrix $G=G(P)$ of signature (*n*, 1) formed by the Lorentzian products $g_{ik}=\langle e_i,e_k\rangle _{n,1}$. The coefficients of *G* off the diagonal have the following geometric meaning.

2.5 $$\begin{aligned} -\langle e_i,e_k\rangle _{n,1}={\left\{ \begin{array}{ll} \displaystyle \cos \frac{\pi }{m_{ik}}&{}\hbox { if}\ \displaystyle \measuredangle (H_i,H_k)=\frac{\pi }{m_{ik}};\\ 1&{} \,\,\text {if} \ H_i,H_k \,\, \text {are parallel};\\ \cosh l_{ik}&{}\hbox { if}\ d_{{{\mathbb {H}}}}(H_i,H_k)=l_{ik}>0. \end{array}\right. } \end{aligned}$$

In [21, pp. 226–227], Vinberg describes an efficient arithmeticity criterion for a hyperbolic Coxeter group $\Gamma$ which we only reproduce in the non-cocompact case. To this end, consider $2G(P)$, and its *cycles* (*of length l*) of the form

2.6 $$\begin{aligned} 2^lg_{i_1i_2}g_{i_2i_3}\cdot \ldots \cdot g_{i_{l-1}i_l}g_{i_li_1}, \end{aligned}$$

with distinct indices $i_j$ in $2G(P)$. Then, $\Gamma$ is arithmetic with field of definition ${{\mathbb {Q}}}$ if and only if all the cycles of $2G(P)$ are rational integers.

In this context, define the field $K(\Gamma ):=\mathbb Q(\{g_{i_1i_2}g_{i_2i_3}\cdot \ldots \cdot g_{i_{l-1}i_l}g_{i_li_1}\})$ of all cycles of *G*(*P*) and call it the *Vinberg field* of $\Gamma$. For $n\ge 3$, the field $K(\Gamma )$ is the smallest field of definition for $\Gamma$, and it is moreover an algebraic number field coinciding with the adjoint trace field of $\Gamma$. As a consequence, the Vinberg field is a commensurability invariant for $\Gamma$ (see [4, Sect. 3]).

Often, we visualise a hyperbolic Coxeter group $\Gamma$ (and its Coxeter polyhedron *P*) in terms of its *Coxeter graph* $\Sigma (\Gamma )$. Each node *i* of $\Sigma (\Gamma )$ corresponds to a generator $s_i$ (and therefore to the vector $e_i$ and the hyperplane $H_i$). Two nodes *i*, *k* are not joined by an edge if the corresponding hyperplanes $H_i$ and $H_k$ are perpendicular. They are joined by a simple edge if the corresponding hyperplanes intersect under the angle ${\pi }/{3}$. The edge carries the weight $m_{ik}\ge 4$, $\infty$, or is replaced by a dotted edge (sometimes with weight $l_{ik}$), if the hyperplanes $H_i,H_k$ intersect under the angle $\pi /m_{ik}$, are parallel, or at the positive hyperbolic distance $l_{ik}$, respectively.

#### Example 2.2

In [9, Thm. 3], all the (finitely many) hyperbolic Coxeter simplices in ${{\mathbb {H}}}^n$, $n\ge 3$, have been classified with respect to commensurability. The six non-arithmetic Coxeter tetrahedra are pairwise incommensurable except for one pair of groups. This pair consists of the 1-cusped Coxeter tetrahedral group  giving rise to a 1-cusped subgroup of index 2.

#### Example 2.3

**Fig. 2:**
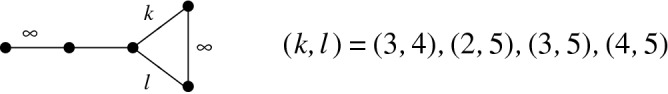
The four non-arithmetic Coxeter pyramids with exactly one ideal vertex in ${{\mathbb {H}}}^3$

Among the 19 non-arithmetic Coxeter pyramids with quadrilateral basis in ${{\mathbb {H}}}^3$, precisely four of them give rise to 1-cusped orbifolds. Their Coxeter graphs are given by Fig. 2. Ignoring their cusp densities, it was shown in [7, Sect. 4.1], that these four orbifolds are incommensurable.

**Fig. 3:**
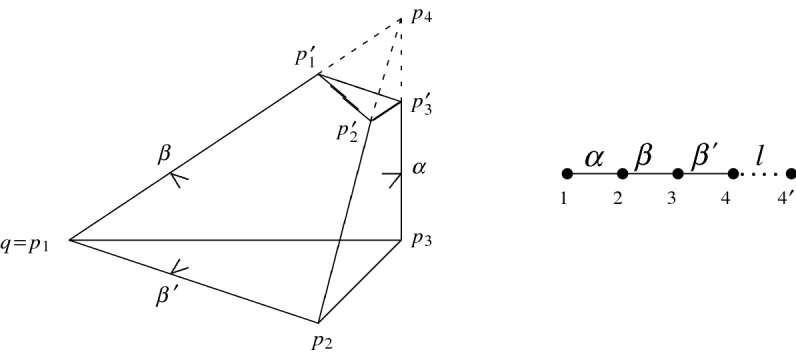
The prism $R'(\alpha ,\beta )\subset {{\mathbb {H}}}^3$ with $0<\alpha +\beta <{\pi }/{2}$ and its Vinberg graph

In contrast to Examples 2.2 and 2.3, there are infinite sequences of Coxeter prisms in ${{\mathbb {H}}}^3$ that give rise to non-arithmetic 1-cusped Coxeter orbifolds. They are at the heart of this work and can be characterised as follows. From a combinatorial-metrical point of view, they arise by polar truncation of an *orthoscheme* $R(\alpha ,\beta )\subset {{\mathbb {H}}}^3$ with $0<\alpha +\beta <\pi /2$. The tetrahedron $R(\alpha ,\beta )$ is an orthogonal tetrahedron of infinite volume bounded by the hyperbolic planes $H_1,\dots ,H_4$ opposite to the vertices $p_1,\dots ,p_4$, say. The planes form one ideal vertex $q=p_1=H_2\cap H_3\cap H_4$ characterised by a Euclidean triangle with angles $\pi /2$, $\beta$, and $\beta '={\pi }/{2}-\beta$, and one ultra-ideal vertex $p_4=H_1\cap H_2\cap H_3$ (represented by a vector $v\in \mathbb R_+^4$ with positive Lorentzian norm) that we cut off by its polar hyperplane $H'_4=\{x\in {{\mathcal {H}}}^3\mid \langle x,v\rangle _{3,1}=0\}$. Associated to $p_4$ is the hyperbolic triangle $R(\alpha ,\beta )\cap H'_4$ with corresponding vertices $p_1'$, $p_2'$, and $p_3'$, and with angles $\pi /2$, $\alpha$, and $\beta$. This triangle is at distance $l=d_{\mathbb H}(p_2,p_2')$ from the opposite triangle in $R(\alpha ,\beta )$. The truncation by means of the hyperbolic plane $H'_4$ leads to a (simplicial) prism $R'(\alpha ,\beta )$ of finite volume that can be described by the *Vinberg graph* according to Fig. 3.

Here, the nodes *i* and $4'$ correspond to the planes $H_i$ and $H'_4$, and two nodes are not joined if the associated planes are Lorentz-orthogonal. For the weight $l=l_{\alpha \beta }$ of the dotted edge corresponding to the length of the common perpendicular of $H_4$ and $H'_4$, an easy computation exploiting the vanishing of the determinant of the Gram matrix of $R'(\alpha ,\beta )$ yields the expression

2.7 $$\begin{aligned} \tanh l_{\alpha \beta }=\tan \alpha \tan \beta . \end{aligned}$$

For the volume of $R'(\alpha ,\beta )\subset {{\mathbb {H}}}^3$, there is a closed formula in terms of $\alpha$, $\beta$, and the Lobachevsky function  as follows (see [10]).2.8Observe that the Lobachevsky function  is odd, $\pi$-periodic and satisfies a certain distribution relation. As an example,2.9which allows one to deduce  for its maximum value. For computations, the series representation2.10with Bernoulli coefficients $B_1={1}/{6}$, $B_2={1}/{30}$, $\ldots$, converges rapidly for small $\omega$ (see [17, App.]).

The formula (2.8) can be derived by integrating Schläfli’s differential formula which expresses the infinitesimal volume change of a non-Euclidean polyhedron in terms of the variation of its dihedral angles (see [10], for example). In particular, when keeping the angle parameter $\beta =\beta _0$ constant, the volume differential of $R'(\alpha ,\beta _0)$ is given by

**Fig. 4:**
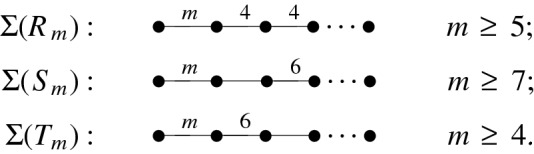
The three sequences $(R_m)$, $(S_m)$, and $(T_m)$ of Coxeter prism groups in ${{\,\textrm{Isom}\,}}{{\mathbb {H}}}^3$

2.11 $$\begin{aligned} d{{\,\textrm{vol}\,}}_3(R'(\alpha ,\beta _0))=-\frac{l_{\alpha \beta _0}}{2}\,d\alpha , \end{aligned}$$

which leads to (2.8) by using ${{\,\textrm{vol}\,}}_3(R'(\beta '_0,\beta _0))=0$ as integration constant. Among the prisms $R'(\alpha ,\beta )\subset {{\mathbb {H}}}^3$ with $0<\alpha +\beta <\pi /2$, there are three distinguished infinite families, indexed by an integer *m*, of Coxeter prisms $R_m$, $S_m$, and $T_m$ with Coxeter graphs given in Fig. 4 (see also Fig. 1 in the Introduction).

By means of Vinberg’s arithmeticity criterion, the 1-cusped Coxeter orbifolds associated to the Coxeter groups given by $R_m$, $S_m$, and $T_m$ are non-arithmetic at least for $m\ge 7$. In the sequel and for convenience, we shall use the same symbol $X_m$ for the Coxeter prism as well as for the associated reflection group and its quotient space.

Our aim is to prove first that the members belonging to a fixed sequence, and secondly, that most pairs from different sequences are incommensurable hyperbolic Coxeter groups. To do this we follow two different paths. The first one is algebraic and based on the study of Vinberg spaces and the relevant results of the first author [3, 4]. We shall see that this approach has limitations. The second path is geometric and based on certain analytic properties of the cusp density function such as strict monotonicity. It leads to a coherent and complete proof of our theorem.

## The Vinberg Space and Commensurability

Let $m\ge 7$, and consider the three sequences $(X_m)$ of non-arithmetic Coxeter prism groups in ${{\,\textrm{Isom}\,}}{{\mathcal {H}}}^3$ depicted in Fig. 4. The subsequent machinery is due to Vinberg, and the new results about commensurability based on it are due to the first author (see [3, 4] and the references therein).

Associated to each group $X_m$ of the sequence $(X_m)$ is the Vinberg field $K(X_m)$ generated by all the cycles $g_{i_1i_2\ldots i_l}$ of the Gram matrix $G(X_m)=(g_{ik})$ of the prism $X_m$ (see Sect. 2.2). Following the description as given in Fig. 3, denote by $e_1,\ldots ,e_4$ and $e_5$ the outer normal unit vectors in $(\mathbb R^4,\langle \,{\cdot }\,,\,{\cdot }\,\rangle _{3,1})$ of the hyperplanes $H_1,\ldots ,H_4$ and $H'_4=:H_5$ bounding $X_m$.

Next, for $1\le i_1,\ldots ,i_k\le 5$, define the vectors

3.1 $$\begin{aligned} v_1=e_1\quad \hbox {and}\quad v_{i_1i_2\ldots i_k}=g_{1i_1}g_{i_1i_2}\cdots g_{i_{k-1}i_k}\,e_{i_k}\,\,. \end{aligned}$$

The $K(X_m)$-space $V(X_m)$ spanned by the vectors $\{v_{i_1i_2\ldots i_k}\}$ according to (3.1) is of dimension 4 and left invariant by the action of the group $X_m$. The restriction of the Lorentzian product to $V(X_m)$ yields a quadratic form $q=q(V(X_m))$ of signature (3, 1). The form *q* and the quadratic space $(V(X_m),q)$ are called the *Vinberg form* and the *Vinberg space* of $X_m$, respectively. The Vinberg field and the Vinberg form (with its discriminant) are closely related to the invariant trace field and the invariant quaternion algebra of a Kleinian group $\Gamma \subset {{\,\textrm{PSL}\,}}(2,{{\mathbb {C}}})$, here given by the rotation subgroup of $X_m$ [14, Thm. 3.1]. In the arithmetic case, these latter algebraic tools form a complete system of commensurability invariants for $\Gamma$.

In the case of arbitrary (cofinite) hyperbolic Coxeter groups, there is the following obstruction to commensurability as proven in [4, Thm.].

### Theorem 3.1

Let $\Gamma _1$ and $\Gamma _2$ be two commensurable hyperbolic Coxeter groups acting on ${{\mathbb {H}}}^n$, $n\ge 2$. Then, their Vinberg fields coincide and the two associated Vinberg forms are similar over this field.

Recall that two quadratic forms $q_1$ and $q_2$, defined on vector spaces $V_1$ and $V_2$ of dimension *m* over a field *K*, respectively, are similar if and only if there exists a scalar $\lambda \in K^*$ such that $(V_1,q_1)$ and $(V_2,\lambda q_2)$ are isometric spaces. Representing the quadratic forms $q_1, q_2$ by means of their bilinear forms with matrices $Q_1,Q_2\in {{\,\textrm{Mat}\,}}(m,K)$, the isometry of $(V_1,q_1)$ to $(V_2,\lambda q_2)$ then means that there is a matrix $S\in {{\,\textrm{GL}\,}}(m,K)$ such that $Q_1=S^t(\lambda Q_2)S$.

In the case of *odd* dimensions $n\ge 3$, the theorem above combined with the Theorem of Hasse–Minkowski produces the following commensurabilitry condition (see [4, Lem. 3.16]).

### Proposition 3.2

(Ratio Test)  For $n\ge 3$ odd, let $\Gamma _1$ and $\Gamma _2$ be two commensurable hyperbolic Coxeter groups acting on ${{\mathbb {H}}}^n$ with Vinberg field *K* and Vinberg forms $q_1$ and $q_2$, respectively. Then, $\det (q_1)\equiv \det (q_2)$ mod $(K^*)^2$.

Let us illustrate the above theorem and examine as far as possible the (in-)commensurability of the non-arithmetic groups $X_m=R_m$, $S_m$, and $T_m$ for $m\ge 7$. In order to establish their Gram matrices $G(X_m)=(g_{ik})$ and compute the Vinberg fields, we determine the weights $l_{mp}=l_{{\pi }/{m},{\pi }/{p}}$, $p=3,4,6$, according to (2.7) and obtain the following results.

3.2 $$\begin{aligned}&\cosh l_{m4}=\frac{\cos (\pi /m)}{\sqrt{\cos (2\pi /m)}},\qquad \cosh l_{m3}=\frac{\cos (\pi /m)}{\sqrt{2\cos (2\pi /m)-1}},\nonumber \\&\cosh l_{m6}=\frac{\sqrt{3}\cos (\pi /m)}{\sqrt{2\cos (2\pi /m)+1}}, \end{aligned}$$

3.3 $$\begin{aligned}&K(R_m)=K(S_m)=K(T_m)=\mathbb Q\biggl (\cos \frac{2\pi }{m}\biggr )=\mathbb Q\biggl (\cos ^2\frac{\pi }{m}\biggr )=:K_m. \end{aligned}$$

The extension degree of $K_m$ equals $[K_m{:}{{\mathbb {Q}}}]=\varphi (m)/2$, where $\varphi (k)$ denotes the Euler totient function counting the positive integers smaller than or equal to *k* that are relatively prime to *k*. Recall that $\varphi (k)$ is not injective since, for example, $\varphi (2k)=\varphi (k)$ for odd *k*.

Next, we determine for each $X_m$ the Vinberg form by following Vinberg’s construction. To this end, we construct the outer normal unit vectors $e_1,\ldots ,e_5$ and choose a basis $v_1,\ldots ,v_4$ for the Vinberg space $V(X_m)$ in the set of vectors defined by (3.1). Their Gram matrix $Q(X_m):=(\langle v_i,v_k\rangle _{3,1})_{1\le i,k\le 4}$ yields the Vinberg form $q(V(X_m))$. For comparison by means of the Ratio Test above, it suffices to compute the determinant of $Q(X_m)$ modulo $K_m^2$. We summarise the computations as follows.

Consider the Coxeter prism $R_m$ as given by the Coxeter graph $\Sigma (R_m)$ depicted in Fig. 4 and with weight $l_{m4}$ according to (3.2). We put $R_m$ in ${{\mathcal {H}}}^3$ in such a way that its outer normal unit vectors are given by

3.4 $$\begin{aligned} \begin{aligned} e_1&=(0,1,0,0),\qquad e_2=\biggl (0,-\cos \frac{\pi }{m},\sin \frac{\pi }{m},0\biggr ),\\ e_3&=\Biggl (0,0,\frac{-1}{\sqrt{1-\cos (2\pi /m)}},\frac{\sqrt{\cot ^2(\pi /m)-1}}{\sqrt{2}}\Biggr ),\\ e_4&=\biggl (\frac{-\cos (\pi /m)}{\sqrt{\cos (2\pi /m)}},0,0,\frac{\sin (\pi /m)}{\sqrt{\cos (2\pi /m)}}\biggr ),\qquad e_5=(1,0,0,0). \end{aligned} \end{aligned}$$

The vectors

3.5 $$\begin{aligned} v_1:=e_1,\quad v_2:=g_{12} e_2,\quad v_3:=g_{12}g_{23}e_3,\quad v_4:=g_{12}g_{23}g_{34}e_4 \end{aligned}$$

form a basis of $V(R_m)$ over $K_m$ and yield the matrix

$$\begin{aligned}Q(R_m)=\begin{pmatrix}1&{}\quad c&{}\quad 0&{}\quad 0\\ c&{}\quad c&{}\quad c/2&{}\quad 0\\ 0&{}\quad c/2&{}\quad c/2&{}\quad c/4\\ 0&{}\quad 0&{}\quad c/4&{}\quad c/4\end{pmatrix},\end{aligned}$$

where

3.6 $$\begin{aligned} c=c_m=\cos ^2\frac{\pi }{m}. \end{aligned}$$

It is not difficult to compute and reduce the determinant of $Q(R_m)$ modulo $K^2_m$ according to

3.7 $$\begin{aligned} \det (Q(R_m))=-\frac{c_m^8}{16}\equiv -1\quad mod \ K_m^2. \end{aligned}$$

Consider the Coxeter prism $S_m$ as given by the Coxeter graph $\Sigma (S_m)$ in Fig. 4 and with weight $l_{m3}$ according to (3.2). The outer normal unit vectors of $S_m$ can be chosen to be $e_1$, $e_2$, and $e_5$ as in (3.4) while the remaining vectors $e_3$ and $e_4$ have to be equal to

3.8 $$\begin{aligned} \begin{aligned} e_3&=\biggl (0,0,\frac{-1}{2\sin (\pi /m)},\frac{\sqrt{2\cos (2\pi /m)-1}}{2\sin (\pi /m)}\biggr ),\\ e_4&=\biggl (\frac{-\cos (\pi /m)}{\sqrt{2\cos (2\pi /m)-1}},0,0,\frac{\sqrt{3}\sin (\pi /m)}{\sqrt{\cos (2\pi /m)-1}}\biggr ). \end{aligned} \end{aligned}$$

It is clear that the vectors $v_1,\ldots ,v_4$ defined by (3.5) form a basis of the Vinberg space $V(S_m)$. For their Gram matrix $Q(S_m)$, one obtains

$$\begin{aligned}Q(S_m)=\begin{pmatrix}1&{}\quad c&{}\quad 0&{}\quad 0\\ c&{}\quad c&{}\quad c/4&{}\quad 0\\ 0&{}\quad c/4&{}\quad c/4&{}\quad 3c/16\\ 0&{}\quad 0&{}\quad 3c/16&{}\quad 3c/16\end{pmatrix},\end{aligned}$$

where $c=c_m$ is given by (3.6). As a consequence,

3.9 $$\begin{aligned} \det (Q(S_m))=-\frac{3}{256}\,c_m^8\equiv -3 \mod K_m^2\,\,. \end{aligned}$$

Consider finally the Coxeter prism $T_m$ as given by the Coxeter graph $\Sigma (T_m)$ in Fig. 4 and with weight $l_{m6}$ according to (3.2). The outer normal unit vectors of $T_m$ can be chosen to be $e_1$, $e_2$, and $e_5$ as in (3.4) so that the remaining vectors $e_3$ and $e_4$ have to be equal to

3.10 $$\begin{aligned} \begin{aligned} e_3&=\biggl (0,0,\frac{-\sqrt{3}}{2\sin (\pi /m)},\frac{\sqrt{2\cos (2\pi /m)+1}}{2\sin (\pi /m)}\biggr ),\\ e_4&=\biggl (\frac{-\sqrt{3}\cos (\pi /m)}{\sqrt{2\cos (2\pi /m)+1}},0,0,\frac{\sin (\pi /m)}{\sqrt{\cos (2\pi /m)+1}}\biggr ). \end{aligned} \end{aligned}$$

It is obvious that the vectors $v_1,\ldots ,v_4$ defined by (3.5) form a basis of the Vinberg space $V(S_m)$. For their Gram matrix

$$\begin{aligned}Q(T_m)=\begin{pmatrix}1&{}\quad c&{}\quad 0&{}\quad 0\\ c&{}\quad c&{}\quad 3c/4&{}\quad 0\\ 0&{}\quad 3c/4&{}\quad 3c/4&{}\quad 3c/16\\ 0&{}\quad 0&{}\quad 3c/16&{}\quad 3c/16\end{pmatrix},\end{aligned}$$

where $c=c_m$ is given by (3.6), one computes

3.11 $$\begin{aligned} \det (Q(T_m))=-\frac{27}{256}c_m^8\equiv -3\quad mod \ K_m^2. \end{aligned}$$

Put together, the calculations leading to (3.7), (3.9), and (3.11) allow us to deduce the following intermediate results in view of Theorem 3.1 and the Ratio Test given by Proposition 3.2.

### First Conclusions

(A) For a *fixed* sequence $(X_m)$, $m\ge 7$, of non-arithmetic Coxeter prism groups given by one of the Coxeter graphs according to Fig. 4, two groups $X_m$ and $X_{m'}$ with $\varphi (m)\ne \varphi (m')$ (and hence different Vinberg fields) are incommensurable. However, if $K(X_m)=K(X_{m'})=:K$, the Ratio Test does not allow us to conclude about their incommensurability since the determinants of the Vinberg forms $q(X_m)$ and $q(X_{m'})$ are equal modulo $K^2$.
(B) Let $k,l\ge 7$. For a group $R_k$ and a group $X_l$ *not* belonging to $(R_m)$, the Ratio Test proves their incommensurability.
(C) Let $k,l\ge 7$. A group $S_k$ and a group $T_l$ are incommensurable if $\varphi (k)\not =\varphi (l)$. In the case $K(S_k)=K(T_l)=:K$, the Ratio Test does not allow us to conclude incommensurability since the determinants of the Vinberg forms $q(S_k)$ and $q(T_{l})$ are equal modulo $K^2$.

## Cusp Density and Commensurability

In the sequel, we provide a complete proof, based on the cusp density invariant, of the theorem as stated in the Introduction for the infinite sequences $(R_m)$, $(S_m)$, and $(T_m)$ given by Fig. 1. To this end, we generalise the context as follows.

Consider the two-parameter family $R'(\alpha ,\beta )\subset \mathbb H^3$ with $0<\alpha +\beta <{\pi }/{2}$ of prisms in ${{\mathbb {H}}}^3$ as depicted in Fig. 3. Each prism results from polar truncation of an orthoscheme $R(\alpha ,\beta )=\bigcap _{1\le i\le 4}H^-_i$ with ideal vertex $q=p_1$ and ultra-ideal vertex $p_4$. For $i\le 3$, denote by $p'_i$ the intersection of $H'_4$ with the geodesic defined by the vertices $p_i$ and $p_4$. By construction, the vertices $p'_1$, $p'_2$, and $p'_3$ describe the hyperbolic triangle $[p'_1p'_2p'_3]$ opposite to the triangular base $[p_1p_2p_3]$ of the prism $R'(\alpha ,\beta )$, and it has angles ${\pi }/{2}$, $\alpha$, and $\beta$ while being orthogonal to $H_1$, $H_2$, and $H_3$.

The triangle $[p'_1p'_2p'_3]\subset H'_4$ is at distance $l_{\alpha \beta }=d_{{{\mathbb {H}}}}(p_3,p'_3)$ from $[qp_1p_2]$. The quantity $l_{\alpha \beta }$ is given by (2.7) and appears as coefficient in Schläfli’s differential according to (2.11).

**Fig. 5:**
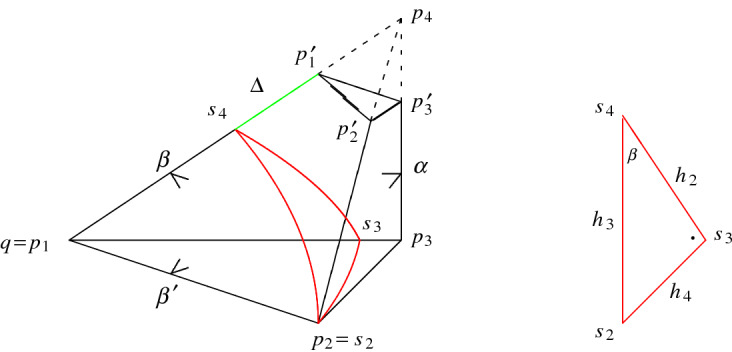
The prism $R'(\alpha ,\beta )\subset {{\mathbb {H}}}^3$ with $0<\alpha +\beta <\pi /2$ such that $\cos \alpha \le \sqrt{2}\sin \beta$ and its cusp triangle $[s_2s_3s_4]$

**Fig. 6:**
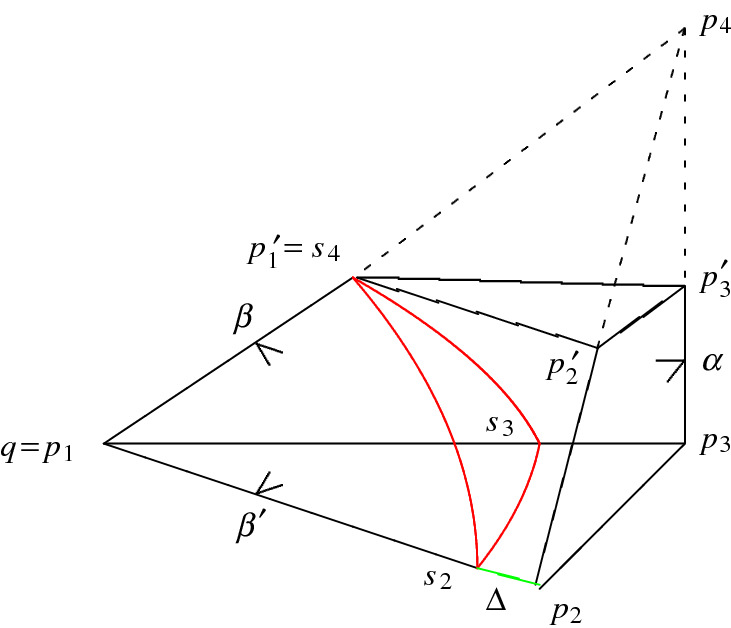
The prism $R'(\alpha ,\beta )\subset {{\mathbb {H}}}^3$ with $0<\alpha +\beta <\pi /2$ such that $\cos \alpha \ge \sqrt{2}\sin \beta$

Our first aim is to derive a formula for the (polyhedral) cusp density4.1Here, $C(\alpha ,\beta )$ is the maximal cusp inside $R'(\alpha ,\beta )$ and results from intersecting the maximal horoball $B_q$ associated to *q* with the prism $R'(\alpha ,\beta )$. Notice that $B_q$ is tangent to the facet(s) closest to *q* but disjoint to the remaining one among all facets not containing *q* in $R'(\alpha ,\beta )$. More precisely, the orthogonality properties reigning in $R'(\alpha ,\beta )$ imply that the horosphere $S_q=\partial B_q$ is either touching $H_1$ at $p_2$ as depicted in Fig. 5, or $H'_4$ at $p'_1$ as depicted in Fig. 6.

Therefore, the size of $C(\alpha ,\beta )$ depends on the geometric position of the planes $H_1,\ldots ,H_4$ and $H'_4$ which can be quantified in terms of the distance $\Delta =\Delta (\alpha ,\beta )$ of $S_q$ to $H_1$ and to $H'_4$, respectively.

**Fig. 7:**
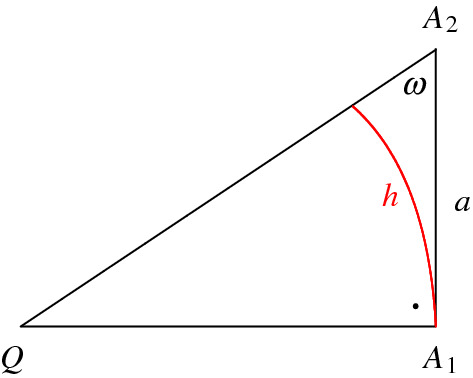
A horocycle in the right-angled triangle $T=[QA_1A_2]$ with ideal vertex *Q*

The following result about horocycle geometry will be useful (see [2, Sect. 4]). Consider a hyperbolic triangle *T* with one ideal vertex *Q*, a right angle at the vertex $A_1$ and the angle $\omega$ at the vertex $A_2$. Let $a=d_{{{\mathbb {H}}}}(A_1,A_2)$, and consider the horocyclic segment of Euclidean length *h* based at *Q* and passing through $A_1$. The situation is depicted in Fig. 7.

### Lemma 4.1

Denote by *h* the Euclidean length of the horocyclic segment in the right-angled triangle $T=[QA_1A_2]$ with ideal vertex *Q*. Let $\omega$ be the angle of *T* at $A_2$ and $a=d_{{{\mathbb {H}}}}(A_1,A_2)$ according to Fig. 7. Then

$$\begin{aligned}h=\cos \omega =\tanh a.\end{aligned}$$

### Proposition 4.2

Let $R'(\alpha ,\beta )\subset {{\mathbb {H}}}^3$ with $0<\alpha +\beta <\pi /2$ be a hyperbolic prism with one ideal vertex *q*. Then, the volume of the maximal cusp neighborhood $C(\alpha ,\beta )$ of *q* in $R'(\alpha ,\beta )$ is given according to the following dichotomy.

(i) $${{\,\textrm{vol}\,}}_3(C(\alpha ,\beta ))=\displaystyle \frac{\cos ^2\alpha \cot \beta }{4}\quad \text {if}~ \cos \alpha \le \sqrt{2}\sin \beta $$;
(ii) $${{\,\textrm{vol}\,}}_3(C(\alpha ,\beta ))=\displaystyle \frac{\sin (2\beta )}{8}\cdot \frac{\cos ^2\alpha }{\cos ^2\alpha - \sin ^2\beta }\quad \hbox { if}\ \cos \alpha \ge \sqrt{2}\sin \beta $$.

### Proof

Start from the representation $R'(\alpha ,\beta )=\bigcap _{1\le i \le 5} H_i^-$ where the plane $H_5$ equals the truncating polar plane $H'_4$ associated to the ultra-ideal vertex $p_4$ of the underlying orthoscheme $R(\alpha ,\beta )$. Since the maximal cusp $C(\alpha ,\beta )$ is either tangent to $H_1$ at $p_2$ or to $H'_4$ at $p'_1$, there are only two possible cases for the relative position of $C(\alpha ,\beta )$, and they are depicted in Figs. 5 and 6, respectively. In both cases, the Euclidean area of the right-angled triangle $[s_2s_3s_4]$ forming the boundary of $C(\alpha ,\beta )$ is given by $(h_4^2/2)\cot \beta$ where $h_4$ denotes the Euclidean length of the segment $[s_2s_3]$ (see Fig. 5). By (2.3), the volume of $C(\alpha ,\beta )$ equals $(h_4^2/4)\cot \beta$. Hence, it remains to determine the quantity $h_4$ in terms of $\alpha$ and $\beta$ as asserted. Accordingly, we distinguish two cases.

*Case *(i)   Suppose that the horosphere $S_q$ centred at *q* touches the plane $H_1$ at $p_2$. By the orthogonality properties of $R'(\alpha ,\beta )$, the dihedral angle $\alpha$ is equal to the angle at $p_3$ in the triangle $[qp_2p_3]$. Hence, by Lemma 4.1, the Euclidean length $h_4$ of the horocyclic segment $[s_2s_3]$ is equal to $\cos \alpha$ implying that ${{\,\textrm{vol}\,}}_3(C(\alpha ,\beta ))=(1/4)\cos ^2\alpha \cot \beta$.

It remains to show that the above assumption holds if $\cos \alpha \le \sqrt{2}\sin \beta$. Since the angle at $s_4$ in the Euclidean triangle $[s_2s_3s_4]$ is equal to $\beta$, the Euclidean length $h_3$ of its hypotenuse is given by

4.2 $$\begin{aligned} h:=h_3=\frac{\cos \alpha }{\sin \beta }. \end{aligned}$$

Observe that $h>1$ since $\alpha +\beta <\pi /2$, and that furthermore $h=\cosh d_{{{\mathbb {H}}}}(p'_1,p'_2)$ by elementary trigonometry for $[p'_1p'_2p'_3]$.

**Fig. 8:**
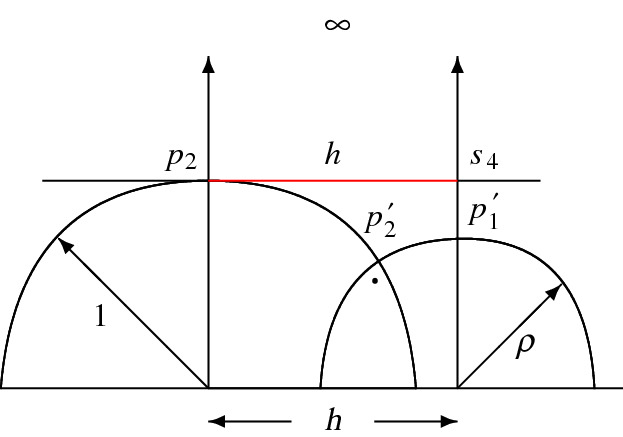
The quadrilateral $[qp'_1p_2p'_2]$ with ideal vertex $q=\infty$

Next, we show that the horosphere $S_q$ does not intersect the plane $H'_4$ which, by the orthogonality properties of $R'(\alpha ,\beta )$, is equivalent to show that $\Delta =d_{{{\mathbb {H}}}}(p'_1,s_4)\ge 0$. For this, we put the Lambert quadrilateral $[qp'_1p_2p'_2]$ in the upper half plane model for ${{\mathbb {H}}}^2$ as follows. Assume without loss of generality that its ideal vertex *q* is $\infty$, and that the horocycle defined by the segment $[s_2s_4]$ and of Euclidean length *h* is at height 1; see Fig. 8. For the distance $\Delta =d_{{{\mathbb {H}}}}(p'_1,s_4)$, we have

$$\begin{aligned}\Delta =\log \frac{1}{\rho },\end{aligned}$$

where $\rho$ denotes the radius of the geodesic semicircle carrying the edge $[p'_1p'_2]$. The geodesic semicircle carrying the edge $[p_2p'_2]$ is of radius 1 and orthogonal to the former one. Furthermore, the centers of these semicircles are at (Euclidean) distance *h* given in (4.2). Hence,

4.3 $$\begin{aligned} \rho ^2+1=h^2=\frac{\cos ^2\alpha }{\sin ^2\beta }. \end{aligned}$$

As a consequence, $\Delta \ge 0$ if and only if $\rho \le 1$, which in turn is equivalent to

$$\begin{aligned}\frac{\cos \alpha }{\sin \beta }\le \sqrt{2}.\end{aligned}$$

This finishes the proof of (i).

*Case *(ii)   The proof is very similar to the one for (i). Suppose that the horosphere $S_q$ centred at *q* touches the plane $H'_4$ at $p'_1$ according to Fig. 6. We determine first the quantity $h=h_3$ giving the Euclidean length of $[s_2s_4]$ in terms of the hyperbolic length of the edge $[p_2p'_2]$ in the quadrilateral $Q=[p_2p'_2p_3p'_3]$ opposite to *q*. The angle at $p_2$ in *Q* equals $\beta '$ while the other angles of *Q* are right ones. Hence, *Q* is a Lambert quadrilateral giving the identity

$$\begin{aligned}\sin \beta =\tanh d_{{{\mathbb {H}}}}(p_2,p'_2)\cdot \tanh d_{{{\mathbb {H}}}}(p_2,p_3).\end{aligned}$$

For the length $d_{{{\mathbb {H}}}}(p_2,p_3)$ of the edge $[p_2p_3]$ in the right-angled triangle $[qp_2p_3]$ with ideal vertex *q* and angle $\alpha$, we get $\tanh d_{{{\mathbb {H}}}}(p_2,p_3)=\cos \alpha$. Putting all this together, we deduce that

4.4 $$\begin{aligned} \cosh d_{\mathbb H}(p_2,p'_2)=\frac{\cos \alpha }{\sqrt{\cos ^2\alpha -\sin ^2\beta }}. \end{aligned}$$

By comparing the situation with case (i) where $h=h_3=\cosh d_{{{\mathbb {H}}}}(p'_1,p'_2)$, we deduce for the Euclidean length $h=h_3$ in case (ii), and by using (4.4), that

4.5 $$\begin{aligned} h=h_3=\frac{\cos \alpha }{\sqrt{\cos ^2\alpha -\sin ^2\beta }}. \end{aligned}$$

Since $h_4=h_3\sin \beta$ with $h_3$ given by (4.5), we conclude that

$$\begin{aligned}{{\,\textrm{vol}\,}}_3(C(\alpha ,\beta ))=\frac{h_4^2}{4}\cot \beta =\frac{\sin (2\beta )}{8}\cdot \frac{\cos ^2\alpha }{\cos ^2\alpha -\sin ^2\beta }.\end{aligned}$$

Finally, it remains to show that the horosphere $S_q$ does not intersect the plane $H_1$ which, by the orthogonality properties of $R'(\alpha ,\beta )$, is equivalent to show that $\Delta =d_{\mathbb H}(s_2,p_2)\ge 0$. Again, consider the quadrilateral $[qp'_1p_2p'_2]$ in the upper half plane model for ${{\mathbb {H}}}^2$ and assume that its ideal vertex *q* is $\infty$, and that the horocycle defined by the segment $[s_2s_4]$ of Euclidean length *h* is at height 1. By performing the exchanges

$$\begin{aligned}p_2\leftrightsquigarrow p'_1,\ \ \quad s_2\leftrightsquigarrow s_4,\end{aligned}$$

Figure 8 gets suitably adapted. As in (4.3), we deduce thatwith the consequence that $\Delta =\log (1/\rho )\ge 0$ if and only if $\cos \alpha \ge \sqrt{2}\sin \beta$. $\square$

### Remark 4.3

Consider the limiting case $\cos \alpha =\sqrt{2}\sin \beta$ in Proposition 4.2. The cusp $C(\alpha ,\beta )$ touches both, the plane $H_1$ at $p_2$ and $H'_4$ at $p'_1$ in the prism $R'(\alpha ,\beta )$ (see Figs. 5 and 6). In the particular instance $\alpha =\pi /k$ with $k\in {{\mathbb {N}}}_{\ge 3}$, the prism $P_k:=R'(\pi /k,\beta )$ appears as building block for each of the two isometric drums that glued together represent a polyhedral model *P* of the (orientable) complement ${{\mathbb {S}}}^3\setminus C_k$ of the sphere ${{\mathbb {S}}}^3$ by the *k*-link chain $C_k$. This construction is due to and nicely illustrated by Thurston [19, Exam. 6.8.1]. A closer look reveals that each drum can be decomposed into 4*k* copies of $P_k$ so that the polyhedron *P* associated to ${{\mathbb {S}}}^3\setminus C_k$ is an ideal one consisting of 8*k* prisms of type $P_k$. As a consequence, the volume of ${{\mathbb {S}}}^3\setminus C_k$ is given bywhere $\beta '=\pi /2-\beta$ by convention. For $k=3$ and $k=4$, the fundamental group of ${{\mathbb {S}}}^3\setminus C_k$ is commensurable to ${{\,\textrm{PSL}\,}}(2,{{\mathcal {O}}}_7)$ and ${{\,\textrm{PSL}\,}}(2,{{\mathcal {O}}}_3)$, respectively (see [19, Examples 6.8.2 and 6.8.3]). The quotient space of ${{\mathbb {S}}}^3\setminus C_k$ by the rotational symmetry group ${{\mathbb {Z}}}_k$ of $C_k$ is obtained by generalised Dehn surgery on the Whitehead link *W*, so thatFinally, we remark that the manifold ${{\mathbb {S}}}^3\setminus W$ is commensurable with the 2-cusped Coxeter orbifold given by the Coxeter pyramid group with graph .

Our next aim is to analyse the cusp density $\delta (\alpha ,\beta _0)$ for *fixed* $\beta _0$ with $0<\alpha +\beta _0<\pi /2$ according to (4.1) and to prove strict monotonicity for the function

4.6 $$\begin{aligned} \delta (\alpha )=\frac{c(\alpha )}{v(\alpha )}:=\frac{{{\,\textrm{vol}\,}}_3(C(\alpha ,\beta _0))}{{{\,\textrm{vol}\,}}_3(R'(\alpha ,\beta _0))}=\delta (\alpha ,\beta _0) \end{aligned}$$

on a suitable interval $[0,\alpha _0]$ with $\alpha _0\in (0,\pi /2)$. We treat the cases $\beta _0=\pi /4$, $\beta _0=\pi /3$, and $\beta _0=\pi /6$ separately in view of the related sequences $(R_m)$, $(S_m)$, and $(T_m)$ given by Fig. 4. We start with the easiest case.

### Lemma 4.4

The density function $\delta (\alpha ,\pi /6)$ is strictly increasing on the interval $[0,\pi /4]$.

### Proof

For $\beta _0=\pi /6$ and $\alpha \in [0,\pi /4]$, we have that $\cos \alpha \ge \sqrt{2}\sin \beta _0$. Hence, by (ii) of Proposition 4.2, the cusp volume of $C(\alpha ,\pi /6)$ is given by

4.7 $$\begin{aligned} c(\alpha )=\frac{\sqrt{3}}{4}\cdot \frac{\cos ^2\alpha }{4\cos ^2\alpha -1}, \end{aligned}$$

which is a strictly increasing function on the interval $[0,\pi /4]$. For the volume $v(\alpha )$ of $R'(\alpha ,\pi /6)$ in the denominator of $\delta (\alpha )=\delta (\alpha ,\pi /6)$, we use Schläfli’s differential (2.11), that is,

$$\begin{aligned}dv(\alpha )=-\frac{l_{\alpha ,\pi /6}}{2}\,d\alpha \end{aligned}$$

to deduce (the classical fact) that $v(\alpha )$ is a strictly decreasing function with respect to $\alpha \in [0,\pi /4]$. As a consequence, $\delta (\alpha )$ is strictly increasing on $[0,\pi /4]$ as claimed. $\square$

For the two remaining cases $\beta _0=\pi /3$ and $\beta _0=\pi /4$, the monotonicity behavior differs but the proof will be uniform.

### Lemma 4.5

- The density function $\delta (\alpha ,\pi /3)$ is strictly increasing on the interval $[0,\pi /7]$. 
- The density function $\delta (\alpha ,\pi /4)$ is strictly decreasing on the interval $[0,\pi /5]$.

### Proof

First, observe that $\cos \alpha \le \sqrt{2}\sin \beta _0$ holds for all $\alpha$ in the case (a) with $\beta _0=\pi /3$ as well as in the case (b) with $\beta _0=\pi /4$. Hence, by (i) of Proposition 4.2, the cusp volume $c(\alpha )$ is given in both cases by

4.8 $$\begin{aligned} c(\alpha )=\frac{\cos ^2\alpha \cot \beta _0}{4}. \end{aligned}$$

In contrast to the function given by (4.7), the numerator $c(\alpha )$ of $\delta (\alpha )$ given by (4.8) is strictly decreasing so that we can not conclude as in the proof of Lemma 4.4. Here, we proceed as follows. Let $l(\alpha )=l_{\alpha \beta _0}$ be the length of the ridge of $\alpha$ which is related to $v(\alpha )$ according $v'(\alpha )=-l(\alpha )/2$. Again, $v(\alpha )$ is a strictly decreasing function. By (2.7), $l(\alpha )$ satisfies the identity

4.9 $$\begin{aligned} l(\alpha )={{\,\textrm{artanh}\,}}{({\tan \alpha \tan \beta _0})}. \end{aligned}$$

We study the sign of the derivative of $\delta (\alpha )$ that can be expressed as

$$\begin{aligned}\delta '(\alpha )=\frac{1}{8}\cdot \frac{\sin (2\alpha )}{v^2(\alpha )}\biggl \lbrace \frac{l(\alpha )\cot \alpha }{2}-2v(\alpha )\biggr \rbrace .\end{aligned}$$

More precisely, we investigate whether the sign of the quantity

4.10 $$\begin{aligned} \Delta (\alpha ):=\frac{l(\alpha )\cot \alpha }{2}-2v(\alpha ) \end{aligned}$$

behaves as claimed according to the cases (a) and (b).

(a) For $\beta _0=\pi /3$, we show that $\Delta (\alpha )>0$ on $[0,\pi /7]$. By (4.9) and using Taylor expansion for ${{\,\textrm{artanh}\,}}t$ for $t:=\sqrt{3}\tan \alpha$, we deduce that

4.11 $$\begin{aligned} \frac{1}{\sqrt{3}}\cdot \frac{l(\alpha )}{\tan \alpha }=\frac{{{\,\textrm{artanh}\,}}t}{t}=1+\frac{t^2}{3}+\frac{t^4}{5}+\frac{t^6}{7}+\cdots . \end{aligned}$$

Obviously, for $t>0$, the function (4.11) is strictly increasing and satisfies

$$\begin{aligned}\lim _{\alpha \rightarrow 0}\frac{1}{2}\cdot \frac{l(\alpha )}{\tan \alpha } =\frac{\sqrt{3}}{2}\approx 0.86602.\end{aligned}$$

Since $-2v(\alpha )$ is strictly increasing as well, and since, by (2.8),we conclude that $\Delta (0)>0$ and the positivity of $\Delta (\alpha )$. In particular, we get $\delta '(\alpha )>0$ on $[0,\pi /7]$.

(b) For $\beta _0=\pi /4$, we proceed in an analogous manner. For $t:=\tan \alpha$, the function

4.12 $$\begin{aligned} \frac{l(\alpha )}{\tan \alpha }=\frac{{{\,\textrm{artanh}\,}}t}{t} \end{aligned}$$

with the expansion as in (4.11) satisfies

$$\begin{aligned}\lim _{\alpha \rightarrow 0}\frac{1}{2}\cdot \frac{l(\alpha )}{\tan \alpha } =\frac{1}{2},\end{aligned}$$

while the value  is equal to Catalan’s constant. Hence, $\Delta (0)\approx -0.41596<0$. Furthermore, we obtain the value

$$\begin{aligned}\Delta \biggl (\frac{\pi }{5}\biggr )=\frac{1}{2}\cdot \frac{l(\pi /5)}{\tan (\pi /5)}-2 v\biggl (\frac{\pi }{5}\biggr )\approx -0.04769<0\end{aligned}$$

with PARI/GP^1^, for example, or by using series representations such as (2.10). As in the case (a), one checks that $\Delta (\alpha )$ is strictly increasing so that both $\Delta (\alpha )<0$ and, by (4.10), $\delta '(\alpha )<0$ on $[0,\pi /5]$. $\square$

We are now ready to provide a uniform proof of the following result announced in the Introduction. This proof is different by nature and allows us to complete the partial conclusions (A) and (C) based on Vinberg’s form as stated at the end of Sect. 3.

### Theorem

For an integer $m\ge 7$, consider the three sequences of non-arithmetic 1-cusped hyperbolic Coxeter 3-orbifolds induced by $(R_m)$, $(S_m)$, and $(T_m)$ according to Fig. 1. Then:

- two distinct elements $X_k$ and $X_l$ belonging to the same sequence are incommensurable;
- each element $R_k$ is incommensurable with any element $X_l$ not belonging to the sequence $(R_m)$;
- the elements $S_k$ and $T_l$ are incommensurable for $k\ge l$.

### Proof

It is an immediate consequence of Proposition 2.1, Lemmas 4.4 and 4.5 that two groups $X_k$ and $X_{l}$ with $k\ne l$ belonging to a *fixed* sequence $(X_m)$, $m\ge 7$, of non-arithmetic Coxeter prism groups as given by Fig. 1 are incommensurable. This proves part (a) of the assertions.

As for part (b), we use the fact that the cusp density function $\delta (\alpha ,\beta _0)$ of the sequence $(R_m)$ is in contrast to those of $(S_m)$ and $(T_m)$ strictly decreasing with respect to $\alpha \in [0,\pi /7]$. Since the limit values at $\alpha =\pi /7$ of the corresponding cusp densities as given by (4.1) and Proposition 4.2 satisfy

$$\begin{aligned}0.48007\approx \delta \biggl (\frac{\pi }{7},\frac{\pi }{4}\biggr )>\delta \biggl (\frac{\pi }{7},\frac{\pi }{6}\biggr )\approx 0.39865,\qquad \delta \biggl (\frac{\pi }{7},\frac{\pi }{4}\biggr )>\delta \biggl (\frac{\pi }{7},\frac{\pi }{3}\biggr )\approx 0.36866,\end{aligned}$$

we conclude that a group belonging to $(R_m)$ is incommensurable with any group belonging either to the sequence $(S_m)$ or to the sequence $(T_m)$. Another verification of this statement has been provided and stated as conclusion (B) at the end of Sect. 3.

In order to prove part (c), it is sufficient in view of Lemma 4.4 to show that

4.13 $$\begin{aligned} \delta \biggl (\alpha ,\frac{\pi }{6}\biggr )>\delta \biggl (\alpha ,\frac{\pi }{3}\biggr )\quad \ \ \text {for all}\quad \alpha \in \biggl (0,\frac{\pi }{7}\biggr ]. \end{aligned}$$

By (4.1) and Proposition 4.2, the inequality (4.13) is equivalent to

4.14 $$\begin{aligned} V(\alpha ):=\frac{v_3(\alpha )}{v_6(\alpha )}>\frac{4\cos ^2\alpha -1}{3}=:C(\alpha )\quad \ \ \text {for all}\quad \alpha \in \biggl (0,\frac{\pi }{7}\biggr ], \end{aligned}$$

where $v_k(\alpha ):={{\,\textrm{vol}\,}}_3(R'(\alpha ,\pi /k))$ for $k=3,6$. Notice that the functions $V(\alpha )$ and $C(\alpha )$ appearing on the left and the right hand side of (4.14) are also defined for $\alpha \in [0,\pi /6]$. Furthermore, by (2.8) and the properties of the Lobachevsky function, we have that

$$\begin{aligned}V(0)=C(0)=1\ \quad \text {and}\quad \ V\biggl (\frac{\pi }{6}\biggr )=C\biggl (\frac{\pi }{6}\biggr )=\frac{2}{3}.\end{aligned}$$

Our strategy is to show that the functions $V(\alpha )$ and $C(\alpha )$ are strictly concave (down) on $(0,\pi /6)$. Obviously, $C(\alpha )$ is strictly concave (down). For the function $V(\alpha )$, we compute the derivative by using Schläfli’s differential expression (2.11). Putting $l_k(\alpha ):=l_{\alpha ,\pi /k}(\alpha )$, we obtain

4.15 $$\begin{aligned} 2 v_6^2(\alpha )V'(\alpha )=l_6(\alpha )v_3(\alpha )-l_3(\alpha )v_6(\alpha ). \end{aligned}$$

By (2.7) we have

$$\begin{aligned}l_k(\alpha )={{\,\textrm{artanh}\,}}{\biggl (\tan \alpha \tan \frac{\pi }{k}\biggr )},\end{aligned}$$

and therefore, $l_3(\alpha )>l_6(\alpha )$ as well as $l_3'(\alpha )>l_6'(\alpha )$ for $\alpha \in (0,\pi /6)$. These properties imply that $v_6(\alpha )>v_3(\alpha )$ by Schläfli’s differential expression, and that

4.16 $$\begin{aligned} d(\alpha ):=2 v_6^2(\alpha )V'(\alpha )<0\ \ \quad \text {for}\quad \ \ \alpha \in \biggl (0,\frac{\pi }{6}\biggr ). \end{aligned}$$

In particular, $V(\alpha )$ is strictly decreasing on $(0,\pi /6)$. By (4.15), and by using again Schläfli’s differential, we obtain for its second derivative that

4.17 $$\begin{aligned} 2 V''(\alpha )=\frac{d'(\alpha )}{v_6^2(\alpha )}-\frac{2 d(\alpha )v_6'(\alpha )}{v_6^3}. \end{aligned}$$

Since the second term in the difference (4.17) is positive by (4.16), $V(\alpha )$ will be strictly concave if $d'(\alpha )<0$ on $(0,\pi /6)$. Similarly to the computation leading to (4.15), and by using the properties of $l_k(\alpha )$ and $v_k(\alpha )$, we obtain that

$$\begin{aligned} d'(\alpha )&=l'_6(\alpha )v_3(\alpha )-\frac{l_6(\alpha )l_3(\alpha )}{2}-l'_3(\alpha )v_6(\alpha )+\frac{l_3(\alpha )l_6(\alpha )}{2}\\&<l'_3(\alpha )[v_3(\alpha )-v_6(\alpha )]<0\qquad \text {for}\quad \alpha \in \biggl (0,\frac{\pi }{6}\biggr ). \end{aligned}$$

Hence, the function $V(\alpha )$ is strictly concave on $(0,\pi /6)$.

Finally, consider the point $\alpha _0=\pi /12\in (0,\pi /6)$. For the function $C(\alpha )$ defined in (4.14), we obtain

$$\begin{aligned}C\biggl (\frac{\pi }{12}\biggr )=\frac{1}{3}\biggl (2\cos \frac{\pi }{6}+1\biggr )=\frac{\sqrt{3}+1}{3}\approx 0.91068,\end{aligned}$$

whereas the value $V(\pi /12)$ can be computed and estimated by using the volume formula (2.8) and the duplication property of the Lobachevsky function  as follows. We obtainSince , we deduce thatBy using Catalan’s constant  and , it follows thatHence, $V(\pi /12)>C(\pi /12)$. This property combined with the facts that $V(\alpha )$ and $C(\alpha )$ are both strictly concave (down) on $(0,\pi /6)$ with identical values at the extremities $\alpha =0$ and $\alpha =\pi /6$ confirms the claims (4.14) and (4.13). $\square$

### Remark 4.6

The proof of part (c) works under the restriction $k\ge l$, only. Indeed, the smooth density functions $\delta (\alpha ,\pi /6)$ and $\delta (\alpha ,\pi /3)$ are strictly increasing with $\delta (\alpha ,\pi /6)>\delta (\alpha ,\pi /3)$ on the interval $(0,\pi /7]$. For their values at $\alpha =0$, we use the cusp density formula (4.1), the identity (2.9) yielding  and Proposition 4.2 in order to conclude thatAs a consequence, for any $\alpha \in (0,\pi /7]$, there is a (unique) $\alpha _*\in (0,\pi /7]$ with $\alpha _*<\alpha$ such that $\delta (\alpha _*,\pi /6)=\delta (\alpha ,\pi /3)$. However, by restricting the real places in $(0,\pi /7]$ to integer submultiples of $\pi$, it might be that the elements $S_k$ and $T_l$ are incommensurable for all integers $k,l\ge 7$. Since there are no counter-examples known to us, we conjecture that the result (c) above holds for all $k,l\ge 7$. However, a proof of this conjecture seems difficult in view of (4.1) and the modest knowledge about the Lobachevsky function.

### Remark 4.7

**Fig. 9:**
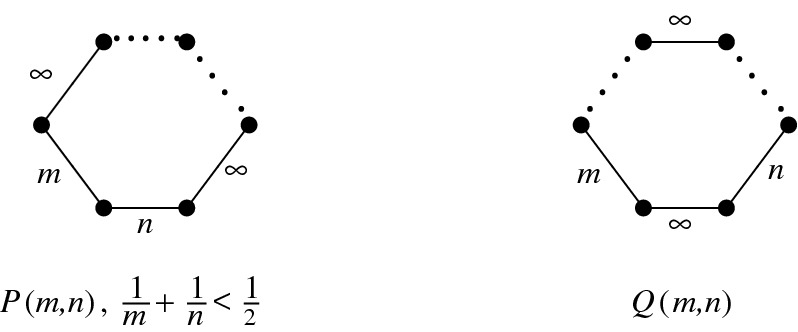
The 1-cusped Coxeter 3-orbifolds associated to *P*(*m*, *n*) and *Q*(*m*, *n*) where $m,n\in {\mathbb {N}}_{\ge 3}$

Similar investigations can be undertaken for other infinite families of non-arithmetic 1-cusped hyperbolic Coxeter 3-orbifolds. For example, there are Coxeter polyhedra *P*(*m*, *n*),  and *Q*(*m*, *n*) in ${{\mathbb {H}}}^3$, described first by Im Hof [8], depending on two integer parameters $m\ge n\ge 3$ and defined by the Coxeter graphs depicted in Fig. 9. For the polyhedron *P*(*m*, *n*), the parameters *m* and *n* have to satisfy the hyperbolicity condition $1/m+1/n<1/2$. Each of the polyhedra has one ideal vertex described by the (disconnected) Coxeter graph  yielding a non-rigid cusp in the associated Coxeter orbifold. The weights belonging to the different dotted edges are easily computable (see [8, Prop. 1.6], for example), and explicit volume formulas can be found in [10]. Finally, by Vinberg’s arithmeticity criterion, the Coxeter groups related to *P*(*m*, *n*) and *Q*(*m*, *n*) are non-arithmetic at least for $m\ge 7$ and $n\ge 3$, and their Vinberg fields coincide and are equal to

$$\begin{aligned}K_{m,n}={{\mathbb {Q}}}\biggl (\cos ^2\frac{\pi }{m},\cos ^2\frac{\pi }{n}\biggr ).\end{aligned}$$

## Data Availability

Data sharing is not applicable to this article as no new data were created or analyzed in this study.

## Footnotes

1 The PARI Group, PARI/GP version 2.11.2, Univ. Bordeaux (2019). http://pari.math.u-bordeaux.fr.
